# Enhancing Research Integrity and Publication Ethics: An Analysis of the Latest International Committee of Medical Journal Editors Recommendations

**DOI:** 10.7759/cureus.56193

**Published:** 2024-03-14

**Authors:** Sankalp Yadav

**Affiliations:** 1 Medicine, Shri Madan Lal Khurana Chest Clinic, New Delhi, IND

**Keywords:** conflicts of interest, authorship, data-sharing, predatory journal, international committee of medical journal editors, icmje

## Abstract

In the ever-evolving landscape of biomedical research and publishing, the International Committee of Medical Journal Editors recommendations serve as a critical framework for maintaining ethical standards. By providing a framework that adapts to technological advancements, the International Committee of Medical Journal Editors recommendations actively shape responsible and transparent practices, ensuring the integrity of scientific inquiry and fostering global collaboration in the ever-evolving landscape of medical publishing. This editorial delves into key aspects of the latest changes in the International Committee of Medical Journal Editors recommendations, focusing on authorship, conflict of interest disclosure, data sharing and reproducibility, medical publishing and carbon emissions, the use of artificial intelligence, and the challenges posed by predatory journals within the realm of open access. It emphasizes the importance of new recommendations, which represent a beacon of ethical guidance in the ever-evolving domain of biomedical research and publishing.

## Editorial

Scientific research forms the backbone of medical progress and knowledge dissemination. As the volume of research output continues to grow, maintaining high standards of integrity and ethical conduct in publication becomes increasingly imperative. The International Committee of Medical Journal Editors (ICMJE) has played a pivotal role in shaping the ethical landscape of medical research through its guidelines for manuscript preparation and publication [1]. This editorial aims to examine the latest ICMJE recommendations, exploring their evolution, impact, and areas for potential improvement.

Evolution of ICMJE recommendations

The ICMJE recommendations, or ‘Recommendations for the Conduct, Reporting, Editing, and Publication of Scholarly Work in Medical Journals', formerly known as the Uniform Requirements for Manuscripts Submitted to Biomedical Journals, initially established in 1978, have undergone several revisions to adapt to the evolving landscape of biomedical research. These recommendations encompass various aspects of publication ethics, including authorship criteria, disclosure of conflicts of interest, and requirements for manuscript preparation. Over the years, these recommendations have become the gold standard for medical journal editors and researchers, providing a framework for transparent and ethical research dissemination. The most recent updated ICMJE recommendations are from January 2024; the important updates are detailed in Table 1 [1].

**Table 1:** Latest updates in the International Committee of Medical Journal Editors recommendations [1]

Topic	Guidance/recommendation	Section
Authorship	Editors should be vigilant about the omission of local researchers from research originating from low- and middle-income countries.	II.A.1
Acknowledgement of artificial intelligence technology in the work	The use of artificial intelligence for writing assistance is acknowledged in the designated (acknowledgment) section. If it was used in a methodology like data collection or analysis or in image or figure generation, then it should be added to the methods section.	I.A.3 and 4 and IV.A.3.d
Use of artificial intelligence in the review of manuscripts	Editors should be aware that the use of artificial intelligence in manuscript processing could compromise confidentiality. Reviewers should be guided about the use of artificial intelligence in review. Besides, reviewers must get prior permission from the journal before using artificial intelligence to complete their reviews.	II.C.2.a and c and II.C.3
Medical publishing and carbon emissions	The journal's governance and link to its owner, like sponsoring society, must be clearly stated. All stakeholders in medical publishing must work toward zero carbon emissions.	II.D.1
Acknowledgment of funding support	Direct support for the project is to be mentioned in the funding statement or disclosure. A distinct or separate statement on the general support for the authors from the institution is to be added.	II.B and IV.A.3.b
Protection of research participants	The authors should have the documentation of the study participants and the data should be readily available at the editor’s request.	II.E
Reference citations	To be made from the published articles, and abstracts to be avoided	IV.A.3.g

Authorship and contributorship

One cornerstone of the ICMJE recommendations is the definition of authorship criteria. The recommendations emphasize that authorship should be based on substantial contributions to the conception, design, execution, or interpretation of the research. This criterion aims to prevent honorary authorship and ensures that individuals who significantly contribute to a study are appropriately recognized.

However, challenges persist in the implementation of these criteria. The pressure to publish in academia sometimes leads to disputes over authorship, and the recommendations may not fully address the nuances of interdisciplinary and collaborative research. Striking a balance between recognizing contributions and avoiding authorship inflation remains a persistent challenge.

The ICMJE recommendations advocate for a transparent and accountable approach to authorship, encouraging detailed documentation of each author's contributions. A clear delineation of roles and responsibilities is paramount, especially in collaborative projects involving multiple institutions or disciplines. The recommendations underscore the importance of all authors being able to defend the integrity and accuracy of the entire work, not just their individual contributions [2].

Additionally, the recommendations address the issue of corresponding authorship, emphasizing the responsibility of the corresponding author to serve as the point of contact for the manuscript, ensure the accuracy of all aspects of the work, and manage communication with the journal during the submission and review process. This underscores the need for effective communication and collaboration among authors to uphold the standards of research integrity. Further, the latest recommendations advise that the editors should send all the correspondence to all the listed authors [1].

Despite the efforts of the ICMJE recommendations, debates persist about the order of authorship and the weight assigned to each author's contributions. In multidisciplinary collaborations, where expertise from various fields converges, establishing a fair and transparent authorship order becomes particularly challenging. Current recommendations are that all the authors should decide on this issue before the work is started and submitted to a journal [1]. The recommendations may benefit from further refinement and contextualization to accommodate the diverse nature of modern scientific research and collaboration.

Although the ICMJE recommendations provide a foundational framework for authorship and contributorship, ongoing discussions within the scientific community are essential to address the evolving challenges in this realm. Collaboration among researchers, institutions, and journal editors is crucial for refining these recommendations to ensure that they continue to serve as a robust and adaptable tool for promoting ethical authorship practices in the ever-evolving landscape of scientific research.

Conflict of interest disclosure

Transparent reporting of conflicts of interest is another crucial aspect of research integrity addressed by the ICMJE recommendations. By requiring authors to disclose financial and non-financial relationships that could potentially bias their work, the recommendations aim to enhance transparency and trust in the scientific literature.

In practice, however, the effectiveness of conflict of interest disclosures can be influenced by the interpretation of what constitutes a significant conflict, and the recommendations may benefit from more explicit definitions in this regard. While financial ties are relatively straightforward, non-financial conflicts can be more nuanced, requiring careful consideration of personal relationships, academic collaborations, and professional affiliations that may influence the research process. These issues were addressed in the latest recommendations [1].

The ICMJE recommendations encourage journal editors to use authors' conflict of interest disclosures as a basis for editorial decisions, enabling readers to critically assess the potential impact of biases on the reported research. However, the implementation of these recommendations varies across journals, and concerns persist regarding the consistency and standardization of disclosure requirements.

Addressing these concerns may involve collaborative efforts between journals, researchers, and professional societies to establish more uniform and detailed reporting standards. Additionally, continuous education and awareness initiatives can help researchers better understand the spectrum of potential conflicts and promote a culture of openness and disclosure within the scientific community.

The rise of industry-sponsored research introduces another layer of complexity to conflict of interest disclosure. While collaboration between academia and industry is essential for advancing scientific knowledge, there is a need for heightened scrutiny to ensure that research outcomes are not unduly influenced by commercial interests [3]. The ICMJE recommendations, in this context, may require periodic reassessment and updates to align with the evolving landscape of research funding and collaboration.

Nevertheless, the recommendations could explore ways to enhance the visibility of conflict of interest disclosures, ensuring that they are readily accessible to readers and incorporated into databases and meta-analyses. This proactive approach would empower the scientific community and the broader public to critically evaluate research findings within the appropriate context of disclosed conflicts.

Data sharing and reproducibility

In recent years, there has been a growing emphasis on data sharing and reproducibility in scientific research. The ICMJE recommendations have incorporated recommendations for data sharing, encouraging authors to make their data available to other researchers, provided ethical and legal considerations are met. There are a number of important developments detailed in the latest ICMJE recommendations related to data sharing [1].

While promoting transparency, the recommendations have sparked debates on issues such as patient privacy, intellectual property, and the burden on researchers to share their data. Striking a balance between open science and protecting the rights of study participants and investigators remains a complex challenge that requires ongoing dialogue and refinement of the recommendations.

One key consideration in the domain of data sharing is the establishment of standardized formats and platforms for sharing diverse datasets. The ICMJE recommendations could further elaborate on best practices for data deposition, specifying acceptable repositories and formats, while also addressing concerns related to the potential misuse or misinterpretation of shared data.

Additionally, the recommendations may benefit from addressing the challenges associated with sensitive data, such as individual patient information. Stricter ethical standards and recommendations for de-identification techniques could enhance the feasibility of sharing even the most sensitive datasets, ensuring that the benefits of data sharing extend to a broader range of research endeavors.

The concept of reproducibility, closely tied to data sharing, emphasizes the ability of researchers to independently validate study findings. While the ICMJE recommendations encourage authors to provide detailed methodologies, challenges still exist in achieving full reproducibility. Considerations such as unique laboratory conditions, proprietary materials, or specialized equipment can pose barriers to exact replication.

To enhance reproducibility, the recommendations could delve into the promotion of detailed and standardized reporting of methodologies, including specific parameters and conditions that are critical for the study's reproducibility. Encouraging the inclusion of comprehensive supplementary materials, such as code scripts and raw data files, would further contribute to the replicability of research findings.

Moreover, fostering a cultural shift toward recognizing the value of replication studies and providing incentives for researchers to conduct and publish them could contribute to strengthening the scientific foundation. Journals adhering to the ICMJE recommendations could play a pivotal role in championing the publication of replication studies, thereby reinforcing the importance of reproducibility in scientific research.

As the scientific community continues to navigate the intricacies of data sharing and reproducibility, the ICMJE recommendations can serve as a catalyst for positive change. By addressing the nuances of diverse datasets, promoting standardized practices, and fostering a culture that values reproducibility, the recommendations have significantly contributed to the robustness and reliability of scientific research. Continuous engagement and collaboration among researchers, publishers, and policymakers will be essential to refine and adapt these recommendations to meet the evolving challenges in the dynamic landscape of data sharing and reproducibility.

Predatory journals and open access

The rise of predatory journals poses a significant threat to the credibility of scientific publishing [4]. The ICMJE recommendations address this issue by promoting ethical practices among journal editors, discouraging researchers from submitting to predatory journals, and advocating for transparency in publication processes [1].

Despite these efforts, the landscape of open-access publishing continues to evolve, necessitating ongoing vigilance and adaptation of the recommendations. As the demand for open access grows, the recommendations could explore additional strategies to empower researchers to distinguish legitimate open-access journals from predatory ones. This might include providing resources, checklists, or recommended practices to aid researchers in making informed decisions about where to submit their work.

The recommendations may also consider addressing the financial challenges associated with open-access publishing. While promoting accessibility to research findings, the article processing charges associated with many reputable open-access journals can be a barrier, particularly for researchers in resource-constrained settings. A more nuanced discussion within the recommendations about balancing the principles of open access with the financial considerations for researchers and institutions could contribute to a fairer and more sustainable open-access publishing landscape.

Furthermore, the ICMJE recommendations could advocate for increased collaboration between institutions, funders, and publishers to establish clear criteria for identifying predatory journals. This collaborative approach could involve the development of a centralized database or registry of reputable journals, offering a reliable resource for researchers seeking to publish in legitimate open-access outlets. The World Association of Medical Editors provides insights regarding the identification of predatory or pseudo-journals [5].

The recommendations may also explore ways to address the issue of deceptive metrics used by predatory journals, which can mislead researchers. Encouraging researchers to rely on established indexing services, impact factors, and peer review processes as markers of journal legitimacy could be emphasized within the recommendations [1].

While the ICMJE recommendations take a commendable stance against predatory publishing practices, the evolving nature of the open-access landscape demands continuous attention. By addressing the financial implications of open access, providing tools for researchers to identify reputable journals, and fostering collaborative efforts to combat predatory practices, the recommendations can further contribute to the preservation of research integrity in an era of expanding open access. Continued collaboration among researchers, institutions, and publishers will be essential to adapting the recommendations and ensuring their efficacy in mitigating the threats posed by predatory journals.

The ICMJE recommendations have undeniably played a crucial role in shaping the ethical landscape of medical research and publication. As the scientific community continues to grapple with evolving challenges, the recommendations must remain dynamic and responsive to emerging issues.

Continuous dialogue among researchers, journal editors, ethicists, and other stakeholders is essential for refining and expanding the ICMJE recommendations. Addressing issues such as authorship disputes, conflict of interest disclosures, data sharing, and predatory publishing requires a collective effort to uphold the highest standards of integrity in scientific research.

In conclusion, the ICMJE recommendations serve as a foundation for ethical conduct in medical publishing, but their effectiveness relies on the commitment of the scientific community to engage in ongoing discussions, share best practices, and adapt to the ever-changing landscape of research and publication.
